# Higher Height, Higher Ability: Judgment Confidence as a Function of Spatial Height Perception

**DOI:** 10.1371/journal.pone.0022125

**Published:** 2011-07-27

**Authors:** Yan Sun, Fei Wang, Shu Li

**Affiliations:** 1 Key Laboratory of Behavioral Science, Institute of Psychology, Chinese Academy of Sciences, Beijing, China; 2 School of Journalism and Communication, Xiamen University, Xiamen, China; University College London, United Kingdom

## Abstract

Based on grounded cognition theories, the current study showed that judgments about ability were regulated by the subjects' perceptions of their spatial height. In Experiment 1, we found that after seeing the ground from a higher rather than lower floor, people had higher expectations about their performance on a knowledge test and assigned themselves higher rank positions in a peer comparison evaluation. In Experiment 2, we examined the boundary conditions of the spatial height effects and showed that it could still occur even if we employed photos rather than actual building floors to manipulate the perceptions of spatial heights. In addition, Experiment 2 excluded processing style as an explanation for these observations. In Experiment 3, we investigated a potential mechanism for the spatial height effect by manipulating the scale direction in the questionnaire. Consequently, consistent with our representational dependence account, the effect of spatial heights on ability judgments was eliminated when the mental representation of ability was disturbed by a reverse physical representation. These results suggest that people's judgments about their ability are correlated with their spatial perception.

## Introduction

Researchers have argued that our mental representations of things we are not able to see or touch may be built out of representations derived from physical experiences with perception [1]–[2]. For example, when speaking or thinking about abstract concepts (e.g., time, power), people often recruit metaphors from spatial concepts [3]. People talk about time or power using spatial words (e.g., a *long* movie, a *high* status). Experimental evidences show that priming spatial information can indeed affect the perception of both time [4]–[5] and power [6]. Broadly speaking, a wide array of research favors the view that human cognition is shaped by sensorimotor experiences [7]–[10].

Ability, another abstract concept, is also often described using spatial language (e.g., a *high*-ability teacher), as are time and power. Some implicit metaphors even reflect the association between ability and spatial heights. For example, a common saying describes an outstanding talent as standing out like a camel in a flock of sheep. The association between ability and spatial heights can also be embodied in everyday experiences. When charts of songs or books are compiled, the winners are listed at the top; when athletes stand on a podium to receive their medals, the champion stands higher than the others. Although spatial positions seem from this to be cues to ability, we have no idea what role they may play in people's thinking about ability. The following arguments and data will try to discover what thinking about seemingly abstract concepts like ability has to do with perception and experience.

Traditional theories about cognition assume that abstract concept representations in the human mind are quite distinct from representations that are formed in modal systems by sensorimotor information. According to these theories, abstract concepts and knowledge only exist in the semantic memory in the form of amodal symbols, which are generally regarded as arbitrary codes transformed by representations in modal systems [2] [11]. As an alternative to such amodal models of mental representation, grounded cognition rejects the representational separation between sensorimotor information and abstract concepts. Instead, grounded cognition proposes that the amodal models of mental representation do not really exist and that the higher level cognitive processes that are responsible for abstract concepts can be directly influenced by sensorimotor information without passing through a redundant change from modal symbols to amodal symbols. Grounded cognition thus argues that the representation of abstract concepts often depends on the representation of sensorimotor information and that metaphors in language are not only communication aids but also the outer modes of internal representations of sensorimotor information [11]. Extensive behavioral and neural evidence from research on cognitive neuroscience, language, social cognition, and developmental psychology supports the grounded cognition view (for a review see Barsalou, 2008).

Grounded cognition theory provides the necessary theoretical background for our predictions, which involve representations of ability and spatial height perception. Based on this grounded cognition perspective, we introduce an interesting possibility: spatial heights may regulate ability judgment. Specifically, when people are primed with a higher rather than a lower spatial height, they will unwittingly change their representations of ability and thus place more confidence on their answers to a general knowledge test.

For this study we designed three experiments to examine the extent to which perceptions of spatial heights regulate ability judgment. In Experiment 1, we sought to determine whether people would increase their self-confidence in a knowledge test when seeing the ground from a higher rather than lower floor. We predicted that such a difference would exist. The purpose of Experiment 2 was twofold. The first goal was to examine the boundary conditions of the phenomenon under investigation. That is, in Experiment 2, we repeated Experiment 1 but employed photos rather than actual building floors to manipulate the participants' perceptions of spatial heights. Based on a grounded cognition perspective, we expected that the activation of spatial heights information using pictures would produce the same effects as obtained in Experiment 1. The second goal of Experiment 2 was to exclude the processing style (i.e., global processing vs. local processing) as an explanation for our observations. We predicted that priming different processing styles would not affect the confidence rating in a subsequent task. Furthermore, in Experiment 3 we explored whether representational dependence (i.e., abstract concepts build on perceptual representation processes) could serve as a conceptual underpinning of the observed effects. We expected that the effect of priming spatial heights on ability judgments would be eliminated or reduced when the mental representation of ability was disturbed by a reverse physical representation.

## Experiment 1

### Methods

#### Ethics Statement

This study was reviewed and approved by the committee for the protection of subjects at the Institute of Psychology, Chinese Academy of Sciences. Written consent was also obtained from each participant before the experiment according to the established guidelines of the committee. This procedure was followed in Experiments 2 and 3 as well.

#### Participants

Ninety-eight undergraduates (46 male, 52 female) with a mean age of 22.6 years (range  = 17–27 yrs) participated in our experiment for course credit. They completed the experiment one at a time.

#### Stimuli and procedure

The place where the participants were tested was the only independent variable we manipulated. Participants were randomly assigned to a meeting room on either the eighth floor or the second floor of a building. The two rooms were exactly same in all respects except for the floor, and the heights from the ground to the windows were respectively 30 meters and 6 meters. Once the participants arrived they were instructed to stand by the appointed window and fix their eyes on the outside ground for three minutes, ostensibly to eliminate emotions. Note that the windows at which the participants stood were located at a consistent position in the different rooms. The weather during the experiment was sunny and the outside visibility was good (more than 10 km). All participants next took the same 10-item general knowledge questionnaire, which involved four knowledge domains (e.g., geography, history, philosophy, sports). The questions were presented in the form of binary choices. An example of the questions is:

Which country is larger? (Check one):

_____ (a) Congo

_____ (b) Zambia

When they had finished all the questions, the participants were asked to estimate how many questions she or he had answered correctly on the questionnaire. The difference between the expected number and the actual number of the correct answers provided an index of the degree of overconfidence. For example, if a participant believed that he had answered six of the questions correctly, when in fact he had gotten only four correct, then he had overestimated his score by two points. This is the most common method for measuring overconfidence [12]. Next, participants answered the following ranking question: Among a random sample of undergraduates, what percentage would you expect to have fewer correct answers than you on the above test? Participants then indicated their mood (“How do you feel right now”) on a 9-point scale (1 =  very unhappy, 9 =  very happy). The experimenter probed participants for suspicion about the true purpose of the exercise and then debriefed them. No participant appeared to be aware of the purpose of the study.

### Results and Discussion

An ANOVA revealed no significant differences in the actual number of correct answers between the higher floor (*M* = 5.51, *SD* = 1.32) and the lower floor (*M* = 5.71, *SD* = 1.33), *F* (1, 96) = .53, *p* = .468, *η_p_^2^ = *.006. However, we found significant differences in the expected number of correct answers, *F* (1, 96) = 9.44, *p* = .003, *η_p_^2^ = *.090. As we predicted, participants on the higher floor reported a higher number of expected correct answers (*M* = 5.94, *SD* = 1.83) than participants on the lower floor (*M* = 4.88, *SD* = 1.56). Furthermore, we calculated the degree of overconfidence for each participant by subtracting the actual number from the expected number. Consistent with our hypothesis, participants on the higher floor expressed more confidence on their answers (*M_difference_* = .43, *SD* = 2.23) than participants on the lower floor (*M_difference_* = −.82, *SD* = 1.96), *F* (1, 96) = 8.70, *p* = .004, *η_p_^2^ = *.083. In fact, twenty-six out of forty-seven participants on the higher floor showed overconfidence (i.e., the difference score was positive) (55%); whereas only thirteen out of fifty-one participants on the lower floor showed overconfidence (25%), χ^2^ = 9.08, *p*<.01. The results of the ranking question also supported our conjecture. Participants on the higher floor marked higher ranks for themselves (*M* = 53.30%, *SD* = 13.56%) than participants on the lower floor (*M* = 46.76%, *SD* = 15.13%), *F* (1, 96) = 5.04, *p* = .027, *η_p_^2^ = *.050. In addition, no significant differences in mood states were reported between the two floor conditions (*M_high_* = 5.30, *SD* = 1.59 vs. *M_low_* = 5.41, *SD* = 1.37), *F* (1, 96) = .15, *p* = .704, *η_p_^2^ = *.002, indicating that the mood of the participants did not change as a function of the building floor.

Consistent with our hypothesis, when participants were assigned to a meeting room on the different floors of a building, the perceptions of spatial heights appear to have regulated their ability judgment. Specifically, when seeing the ground from a higher floor, people increased their expectations about their performance on a knowledge test and assigned themselves higher rank positions in a peer comparison evaluation.

## Experiment 2

Experiment 1 revealed an association between higher spatial height and higher ability inferences. However whether actual height is necessary to produce these effects was unclear. According to grounded cognition, modal models of mental representation that are tied to a perceptual basis not only process sensorimotor information when a stimulus appears but also reenact the sensorimotor state when the stimulus disappears [1]. In Experiment 2, we therefore hypothesized that spatial heights information that was primed by pictures would also be able to affect ability judgments. The other goal of Experiment 2 was to rule out processing style as an explanation for the observations of Experiment 1. Since we assigned participants in Experiment 1 to a meeting room on the different floors to manipulate the perception of vertical space, it could be argued that the higher view might have led to a more global processing style compared to a more local processing style on the lower floor. Indeed, this variation in processing style seemed to present an additional, and potentially confounding, factor. We thus attempted to disentangle this issue in Experiment 2.

### Methods

#### Participants

One hundred and one undergraduates (36 male, 65 female) with a mean age of 19.8 years (range  = 18–22 yrs) participated in Experiment 2 for course credit. They completed the experiment in a class setting in groups.

#### Stimuli and procedure

To manipulate their perceptions of spatial height, the participants were asked to rate photos before the knowledge test. The locations where the photos were taken were either from the eighth floor or the second floor of the same building as in Experiment 1. Note that when taking these photos, our camera always focused on the ground to highlight the vertical altitude. The photos taken at the two different floors thus captured the same objects. The weather at the time when the photos were taken was sunny and the outside visibility was good (more than 10 km). To manipulate the processing style, we masked the background areas in all the photos with gray-black color using Photoshop soft. As a result, the photos were only clear around the central object portions of the masked photos. In this way the attention of the participants was focused on the central object because the backgrounds were hard to see. Thus these masked photos should lead to a more local processing style, compared with normal photos. Consequently, this was a 2 (high spatial height vs. low spatial height) ×2 (local processing style vs. global processing style) between-subject experimental design.

The participants were randomly divided into four groups of approximately 25 each. After that, the four groups were directed to four separate but similar classrooms. Each group responded to one photo condition. As a cover story, participants were informed that the experiment consisted of two unrelated parts: the first involved photo rating and the second was a general knowledge test. Next, the photos appeared on the classroom projection screen, and the questionnaires were presented to all participants at the same time. In the first part of the questionnaire, as a manipulation check, the participants rated the weather when these photos were taken (1 =  very unclear, 9 =  very clear) and their liking for these photos (1 =  not at all, 9 =  very much) and estimated the height from the ground to the location where these photos were taken. In the second part of the questionnaire, the materials and procedures were identical to those used in Experiment 1. At the end, the experimenter probed the participants for suspicion about the purpose of the experiment and then debriefed them. No participant appeared to be aware of the purpose of the study.

#### Manipulation check

As we had expected, participants in the high height photo group estimated a higher height from the ground to the location where these photos were taken (*M* = 38.25 m, *SD* = 39.12 m) than participants in the low height photo group (*M* = 9.42 m, *SD* = 10.38 m), *F* (1, 99) = 24.95, *p*<.001. However no significant differences were found in the weather condition between the high height photo group (*M* = 6.75, *SD* = 1.48) and the low height photo group (*M* = 7.22, *SD* = 1.26), *F* (1, 99) = 2.99, *p* = .087. Similarly, no significant differences were found in the participants' liking for the photos between the high height photo group (*M* = 5.17, *SD* = 1.26) and the low height photo group (*M* = 5.16, *SD* = 1.45), *F* (1, 99) = .001, *p* = .971.

### Results and Discussion

A 2×2 ANOVA on the actual number of correct answers revealed that the main effects were not significant for either spatial height (*M_high_* = 5.77, *SD* = 1.41; *M_low_* = 5.98, *SD* = 1.49, *F* (1, 97) = .62, *p* = .432, *η_p_^2^ = *.006) or processing style (*M_global_* = 5.68, *SD* = 1.46; *M_local_* = 6.02, *SD* = 1.43, *F* (1, 97) = 1.44, *p* = .232, *η_p_^2^ = *.015), and no significant interaction was found between them, *F* (1, 97) = .01, *p* = .946, *η_p_^2^<*.001.

As we predicted, a 2×2 ANOVA on the expect number of correct answers revealed a significant main effect for spatial height (*M_high_* = 5.62, *SD* = 1.75; *M_low_* = 4.43, *SD* = 1.40, *F* (1, 97) = 14.20, *p*<.001, *η_p_^2^ = *.128) but no significant effect for either the main effect of processing style (*M_global_* = 5.02, *SD* = 1.68; *M_local_* = 5.05, *SD* = 1.72, *F* (1, 97) = .02, *p* = .885, *η_p_^2^<*.001) or the interaction between processing style and spatial height condition, *F* (1, 97) = .38, *p* = .539, *η_p_^2^ = *.004.

Furthermore, we calculated the degree of overconfidence for each participant by subtracting the actual number from the expected number. The results turned out to be consistent with our expectations in that participants in the high height photo group expressed more confidence in their answers (*M_difference_* = −.15, *SD* = 2.16) than participants in the low height photo group (*M_difference_* = −1.55, *SD* = 1.72). An ANOVA on the score difference of overconfidence revealed a significant main effect for spatial height (*F* (1, 97) = 13.33, *p*<.001, *η_p_^2^ = *.121) but showed non-significant effects for either processing style (*F* (1, 97) = 1.01, *p* = .317, *η_p_^2^ = *.010) or the interaction between processing style and spatial height condition (*F* (1, 97) = .21, *p* = .652, *η_p_^2^ = *.002).

Similarly, an ANOVA on the ranking results supported our hypothesis. The spatial height main effect was significant, *F* (1, 97) = 4.44, *p* = .038, *η_p_^2^ = *.044, and participants in the high height photo group chose higher ranks for themselves (*M* = 53.46%, *SD* = 15.32%) than participants in the low height photo group (*M* = 47.55%, *SD* = 12.67%). However neither the main effect of processing style, *F* (1, 97) = .06, *p* = .813, *η_p_^2^ = *.001 nor the interaction between them, *F* (1, 97) = .19, *p* = .660, *η_p_^2^ = *.002 was significant.

In addition, an ANOVA on the mood states revealed non-significant effects for spatial height (*F* (1, 97) = 2.79, *p* = .098, *η_p_^2^ = *.028), for processing style (*F* (1, 97) = .01, *p* = .927, *η_p_^2^<*.001), and for the interaction between them (*F* (1, 97) = 1.21, *p* = .274, *η_p_^2^ = *.012).

The results of Experiment 2 suggest that actual height is not necessarily required to produce the effects we observed in Experiment 1 in that a perception of spatial height, as primed by photos, can also influence judgments about ability. In addition, the alternative processing style account, namely that people at higher positions have more confidence in their ability because their higher view might result in a more global rather than local processing style, can be ruled out on the basis of the data from Experiment 2, which confirmed that self-confidence in the participants' judgment did not change as a function of the processing style.

## Experiment 3

**Figure 1:**
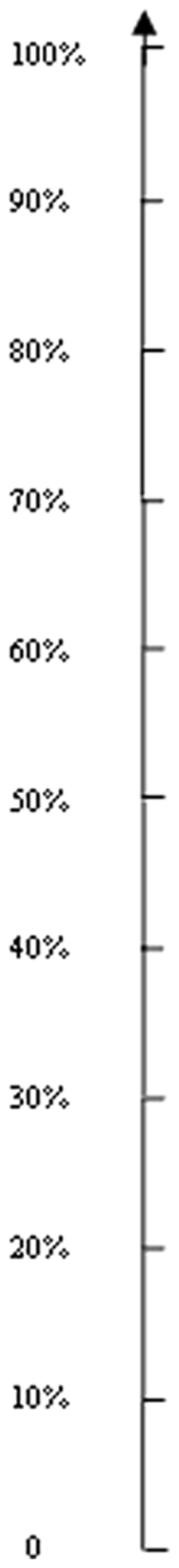
The up-version of the response scale for the ranking question in Experiment 3.

**Figure 2:**
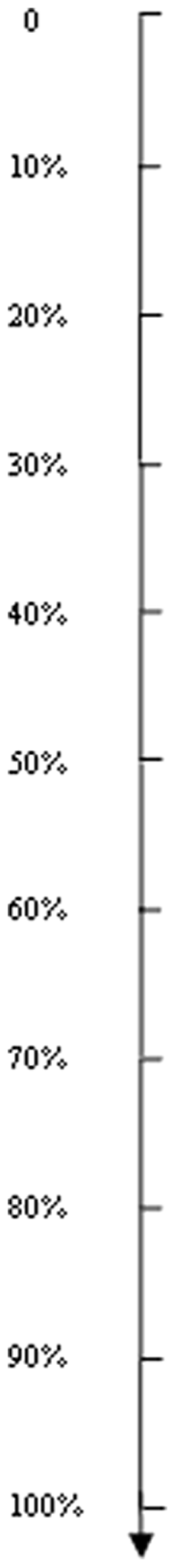
The down-version of the response scale for the ranking question in Experiment 3.

Experiments 1 and 2 supported our hypothesis that a perception of spatial height would regulate ability judgment, but they yielded no information about the underlying reason for the height manipulation effect. Based on grounded cognition, we proposed representational dependence as a mechanism to account for this effect. Specifically, we argued that because ability is mentally represented as a vertical dimension, thinking about ability may involve a mental simulation of space that can be influenced by vertical spatial information. Experiment 3 was designed to determine the reasonability of our representational dependence account. According to the representational dependence concept, representation of ability should be spatially organized along a mental vertical line along which a continuum of increasing ability is perceived. If this is the case, if ability were to be described as increasing in a downward direction, rather than an upward one by the rating scale of the questionnaire (see Figures 1 and 2), we conjectured that the inner mental representation of ability would be disturbed by the reverse outer physical representation of it, and thus the effect of spatial heights on ability judgments would be eliminated or reduced.

### Methods

#### Participants

Ninety-seven undergraduates (31 male, 66 female) with a mean age of 19.6 years (range  = 18–22 yrs) participated in Experiment 3 for course credit. They completed the experiment in a class setting in groups.

#### Stimuli and procedure

In Experiment 3, we used the same materials developed in Experiment 2, but with several modifications. First, since in Experiment 2, the ability judgments did not change as a function of the processing style, we simply employed normal photos to prime the perception of spatial heights, and the masked photos were not used again in Experiment 3. Second, when participants were asked to mark their positions on the ranking question, the rating scale was arranged vertically rather than horizontally. More important, participants randomly responded either to the up-version scale or to the down-version scale, as in Figures 1 and 2. In addition, to demonstrate clearly that the effects were independent of their arousal levels, all participants at the end of the questionnaire indicated their arousal levels during the experiment on a 9-point scale (1 =  sleepiness, 9 =  high arousal).

Experiment 3 also followed the procedure developed in Experiment 2, except that participants were randomly divided into two rather than four groups. As a result, 46 participants saw the low height photos, and the other 51 participants saw the high height photos. In each photo condition, participants were randomly presented with questionnaires that had either the up-version scale or the down-version scale in the ranking question. Thus, this was a 2 (high spatial height vs. low spatial height) ×2 (up-version scale vs. down-version scale) between-subject experimental design. Note that in the questionnaire, the ranking question appeared later than the number estimation question for the correct answers, so the participants' confidence in their number estimation would have nothing to with the scale direction manipulation. Thus, for the expected number of correct answers, we predicted that a significant main effect of spatial height would exist, as in Experiment 2. For the ranking results, however, we predicted a significant interaction between spatial height and the scale version, that is, that the spatial height effect would appear in the up-version scale but not in the down-version scale.

#### Manipulation check

As we expected, participants in the high height photo group estimated a higher height from the ground to the location where these photos were taken (*M* = 43.73 m, *SD* = 46.81 m) than did participants in the low height photo group (*M* = 11.70 m, *SD* = 14.62 m), *F* (1, 95) = 19.78, *p*<.001. However, no significant differences were found in the weather condition between the high height photo group (*M* = 6.25, *SD* = 1.25) and the low height photo group (*M* = 6.74, *SD* = 1.37), *F* (1, 95) = 3.31, *p* = .072. Similarly, no significant differences in liking the photos were found between the high height photo group (*M* = 4.86, *SD* = 1.02) and the low height photo group (*M* = 5.00, *SD* = 1.12), *F* (1, 95) = .40, *p* = .528.

### Results and Discussion

A 2×2 ANOVA on the actual number of correct answers revealed that the main effects were not significant for either spatial height (*M_high_* = 6.02, *SD* = 1.16; *M_low_* = 5.83, *SD* = 1.23, *F* (1, 93) = .58, *p* = .448, *η_p_^2^ = *.006) or scale direction (*M_up_* = 5.86, *SD* = 1.35; *M_down_* = 6.00, *SD* = 1.01, *F* (1, 93) = .38, *p* = .537, *η_p_^2^ = *.004), and no significant interaction was detected between them, *F* (1, 93) = 1.22, *p* = .272, *η_p_^2^ = *.013.

As we predicted, a 2×2 ANOVA on the expected number of correct answers revealed a significant main effect for spatial height (*M_high_* = 5.47, *SD* = 1.39; *M_low_* = 4.48, *SD* = 1.85, *F* (1, 93) = 8.75, *p* = .004, *η_p_^2^ = *.086), but neither the main effect of scale direction (*M_up_* = 4.80, *SD* = 1.98; *M_down_* = 5.21, *SD* = 1.32, *F* (1, 93) = 1.43, *p* = .235, *η_p_^2^ = *.015) nor the interaction between scale direction and spatial height condition, *F* (1, 93) = .60, *p* = .439, *η_p_^2^ = *.006 was significant.

Following Experiments 1 and 2, we calculated the degree of overconfidence for each participant. Results showed that participants in the high height photo group expressed more confidence in their answers (*M_difference_* = −.55, *SD* = 1.85) than did participants in the low height photo group (*M_difference_* = −1.35, *SD* = 2.07). An ANOVA on the score difference of overconfidence revealed a marginally significant effect for spatial height (*F* (1, 93) = 3.89, *p* = .052, *η_p_^2^ = *.040) but showed non-significant effects for either scale direction (*F* (1, 93) = .37, *p* = .545, *η_p_^2^ = *.004) or the interaction between scale direction and spatial height condition (*F* (1, 93) = .001, *p* = .974, *η_p_^2^<*.001).

An ANOVA on the ranking question revealed that the main effects were not significant for either spatial height (*F* (1, 93) = 1.35, *p* = .248, *η_p_^2^ = *.014) or scale direction (*F* (1, 93) = .03, *p* = .858, *η_p_^2^<*.001). Consistent with our prediction, however, a significant interaction was found between them, *F* (1, 93) = 5.18, *p* = .025, *η_p_^2^ = *.053. Specifically, although participants in the high height photo group marked higher ranks for themselves than did participants in the low height photo group for the up-version scale (*M_high_* = 57.20%, *SD* = 13.16%; *M_low_* = 47.92%, *SD* = 13.51%, *t* (47) = −2.44, *p* = .019 ), no difference was found between the high height photo group and the low height photo group for the down-version scale (*M_high_* = 51.54%, *SD* = 13.47%; *M_low_* = 54.55%, *SD* = 12.90%, *t* (46) = .79, *p* = .44 ).

In addition, an ANOVA on the mood states of the participants revealed non-significant effects for spatial height (*F* (1, 93) = 3.80, *p* = .054, *η_p_^2^ = *.039), for scale direction (*F* (1, 93) = .84, *p* = .361, *η_p_^2^ = *.009), and for the interaction between them (*F* (1, 93) = .79, *p* = .377, *η_p_^2^ = *.008). An ANOVA on the arousal levels revealed non-significant effects for spatial height (*F* (1, 93) = .65, *p* = .423, *η_p_^2^ = *.007), for scale direction (*F* (1, 93) = .06, *p* = .815, *η_p_^2^ = *.001) and for the interaction between them (*F* (1, 93) = 1.19, *p* = .279, *η_p_^2^ = *.013).

Experiment 3 provided further evidence that the perception of spatial height can influence judgments of ability. More importantly, Experiment 3 supported representational dependence as a conceptual underpinning for the observed effects. That is, when the inner mental representation of ability was disturbed by a reverse outer physical representation of it (i.e., down-version scale), the effect of spatial height on ability judgments disappeared; when the inner mental representation of ability was consistent with its outer physical representation (i.e., up-version scale), the spatial height effect appeared. Interestingly, Experiment 3 found a moderating effect of scale direction only in the ranking question, but not in the estimated number of correct answers. The participants had not seen the rating scale when they answered the number estimation question because the ranking question was presented after the number estimation question in the questionnaire. Thus, the difference in the results between these two questions seems to be a valid one which further validates the sensitivity of our scale direction manipulation. In addition, Experiment 3 clearly established the independence of these height effects from mood states and arousal levels.

From this series of experiments, we discovered that judgments about ability are regulated by perceptions of spatial heights. In Experiment 1, we showed that people increased their self-confidence about a knowledge test after seeing the ground from a higher rather than lower floor. In Experiment 2, we examined the boundary conditions of the spatial height effects and showed that it could still occur even if we employed photos rather than actual building floors to manipulate the perceptions of spatial heights. Experiment 2 also excluded processing style as an explanation for our observations. In Experiment 3, we investigated a potential mechanism for the spatial height effect by manipulating the scale direction in the questionnaire. Consequently, and consistent with our representational dependence account, the effect of spatial height on the participants' judgments about their ability was eliminated when the mental representation of ability was disturbed by its reverse physical representation. To our knowledge, the present study is the first to suggest that people's ability judgment is not independent of spatial perception. These results are consistent with theories of grounded cognition that have revealed interesting correspondences between concrete physical experience and abstract cognition [13]–[18].

Most relevant to the current study, Meier, Hauser, Robinson, Friesen, and Schjeldahl (2007) found that inferences related to divinity relied on vertical representation processes [19]. Schubert (2005) showed that thinking about power involved a mental simulation of space and could be interfered with by a perception of vertical differences [6]. These findings, along with the data reported in the current study, suggest that spatial height may offer a communal modal system to support multiple and distinct concept representations. This communal representation argument is consistent not only with grounded cognition but also with metaphor representation processes. Lakoff and Johnson (1999) pointed out that the human conceptual system is often deemed to be structured around only a small set of fundamental experiential concepts, such as space and actions, and all other abstract concepts must be understood and structured through metaphorical mappings from these fundamental concepts [2]. Our results highlight the role of space as a fundamental concept and thus provide evidence for such a representational dependence view.

Previous researchers found that body height may play a role in judging other persons' ability. That is, people tend to regard the ability of taller persons as higher [20]–[22]. However, rather than investigating body height, the present study developed a new connection between the ability and perceptions of the spatial height of the occupied site.

In the current study, we clearly showed that ability judgment, as a psychological variable, can be affected by perceptions of spatial height. Interestingly, Proffitt (2006) showed conceptually similar effects in the reverse direction, that is some psychological factors impacted spatial perception. For example, Proffitt found that people's goals in fluenced their distance perceptions [23]. Both the current finding and Proffitt's observation challenge the view of traditional cognition theories that abstract knowledge representations are separate from modal systems formed by sensorimotor information and also help to sharpen the understanding of the fundamental reciprocity that occurs between perceptual interference and knowledge accessibility.

In addition, in view of the fact that controlling people's perceptions of spatial heights should be a relatively inexpensive and nonintrusive way to restore the representation of ability, our findings have important applied implications for promoting people's self confidence in previously unexplored ways.
